# Verification of the *Saccharina japonica* Translocon *Tic20* and its Localization in the Chloroplast Membrane in Diatoms

**DOI:** 10.3390/ijms20164000

**Published:** 2019-08-16

**Authors:** Zhihang Chen, Xiuliang Wang, Shuang Li, Jianting Yao, Zhanru Shao, Delin Duan

**Affiliations:** 1 Key Laboratory of Experimental Marine Biology, Center for Ocean Mega-Science, Institute of Oceanology, Chinese Academy of Sciences, Qingdao 266071, China; 2 Laboratory for Marine Biology and Biotechnology, Qingdao National Laboratory for Marine Science and Technology, Qingdao 266071, China; 3 University of Chinese Academy of Sciences, Beijing 100093, China

**Keywords:** Tic20, EF-hand, *Phaeodactylum tricornutum*, *Saccharina japonica*, subcellular localization, transmembrane topology

## Abstract

Tic20 is an important translocon protein that plays a role in protein transport in the chloroplast. The sequence of Tic20 was determined in the lower brown alga *Saccharina japonica*. Structural analysis of SjTic20 revealed a noncanonical structure consisting of an N-terminal non-cyanobacterium-originated EF-hand domain (a helix-loop-helix structural domain) and a C-terminal cyanobacterium-originated Tic20 domain. Subcellular localization and transmembrane analysis indicated that SjTic20 featured an “M”-type N_in_-C_in_-terminal orientation, with four transmembrane domains in the innermost membrane of the chloroplast in the microalga *Phaeodactylum tricornutum*, and the EF-hand domain was entirely extruded into the chloroplast stroma. Our study provides information on the structure, localization, and topological features of SjTic20, and further functional analysis of SjTic20 in *S. japonica* is needed.

## 1. Introduction

Translocons at the outer/inner envelope (OE/IE) membranes of chloroplasts (designated as the TOC and TIC complexes, respectively) are hetero-oligomeric protein complexes, regarded as the major machinery responsible for the transport of nuclear-encoded preproteins into chloroplasts [1,2]. Tic20 is an important TIC complex that localizes to the inner membrane of chloroplasts [3,4,5]. This complex is believed to be the channel for preprotein translocation [6,7,8]. The canonical Tic20 protein contains four transmembrane α-helixes with N- and C-termini oriented into the stroma (N_in_–C_in_), characterized by the “M”-like membrane spanning model [9]. Reduced expression of Tic20-I can result in albinism, pale leaves, defects in preprotein import into chloroplasts, and significant growth defects in *Arabidopsis thaliana* [7,10,11].

Little is known regarding the localization and function of Tic20 in lower organisms, especially organisms that possess secondary plastids. Tic20 was characterized by being localized to the innermost plastid membrane via the prediction of 4 α-helixes in *Bigelowiella natans* [12], *Cyanophora paradoxa* [13], *Ectocarpus siliculosus*, and *Fucus vesiculosus* [14], but there is no experimental evidence to support this prediction. van Dooren et al. [15] found that *Toxoplasma gondii* Tic20 localized to the innermost apicoplast membrane, and its transmembrane α-helixes exhibited N_in_–C_in_ topology, consistent with the topology observed in higher plants; downregulated expression of TgTic20 caused deficiency in growth and impairment of apicoplast preprotein import. Our previous SNP-based quantitative trait locus (QTL) mapping showed that 14 predicted tandem repeat *Tic20s* were potential candidate genes associated with both blade length and width in *Saccharina japonica* [16]. Therefore, it is necessary to verify the locations and functions of these genes in kelp.

Subcellular localization and transmembrane topology are crucial for the ability of membrane proteins to fulfil diverse cellular functions [17,18]. Although numerous prediction tools are available, the common divergence of the structures and localization of proteins has made predictions of localization and topology highly complicated in organisms that possess secondary plastids [2,19]. Due to the lack of a system for subcellular localization in brown macroalgae, we adopted a microalgal system to ascertain the location of SiTic20s in *Phaeodactylum tricornutum*, which shares a similar structure with kelp, containing four plastid-surrounding membranes [19,20]. Moreover, this diatom was an ideal model for heterologous expression of genes from Bacillariophyta (*Odontellasinensis*), Cryptophyta (*Guillardia theta*), Chlorophyta (*Chlamydomonas reinhardtii*) [20,21,22], etc.

To characterize the features and localization of SjTic20, we examined the structure, subcellular localization, and topological features of this complex in the microalga *P. tricornutum*. Our study will not only verify the function of SjTic20 in the growth of *S. japonica*, but also be significant in understanding the role of this complex in protein transport in the plastids of lower plants.

## 2. Results

### 2.1. Features of SjTic20-14

**Figure 1:**
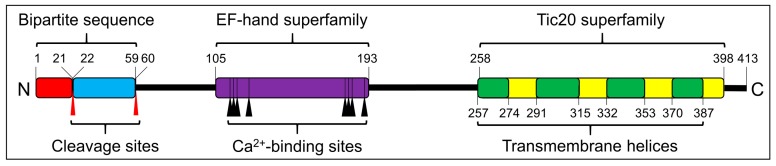
Schematic of the predicted SjTic20-14 protein structure. Signal peptide (red box), transit peptide-like sequence (blue box), EF-hand domain (violet box), Tic20 domain (yellow box), transmembrane region (green box), cleavage sites (red arrows), Ca^2+^-binding sites (black arrows).

After searching transcriptome databases, we identified *SjTic20-14* as our target gene. The full sequence of *SjTic20-14* consisted of 1835 bp (NCBI accession no. MK440682), including a 48 bp 5’- untranslated region (UTR), a 545 bp 3’-UTR, and a 1242 bp coding sequence (CDS), which encoded 413 aa (predicted MW 44056 Da) (Figure 1; Figure S1). Structural prediction of SjTic20-14 indicated one N-terminal bipartite targeting sequence (BTS) with a 1-21 aa signal peptide (SP) and a 22-58 aa transit peptide (TP). The EF-hand domain (105-193 aa) with eight Ca^2+^-binding sites (aa 113, 115, 117, 124, 180, 182, 184, and 191) was followed by a Tic20 domain containing four predicted transmembrane domains (TMDs) (aa 257-247, 291-315, 332-353, and 370-387) (Figure 1; Figure S1). Subcellular localization and transmembrane topology analyses showed that the protein exhibited exclusive chloroplast membrane localization with the “M”- or “W”-type membrane-spanning model in *S. japonica* (Figure S1). In addition, the expression level of *SjTic20-14* increased gradually with organism development (Figure S2).

**Figure 2:**
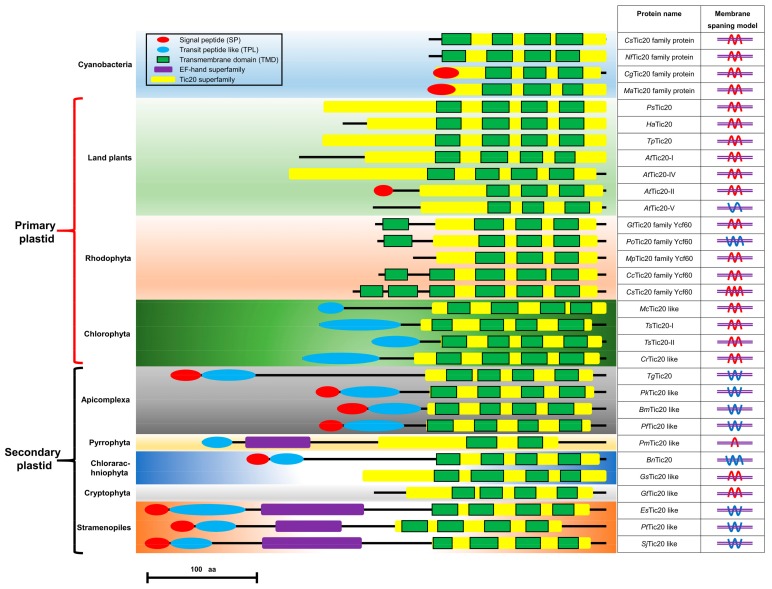
Schematic depiction of the structures and membranes of Tic20-like proteins in different organisms. SP: signal peptide (red oval), TPL: transit peptide-like sequence (blue oval), TMD: transmembrane domain (green box), EF-hand domain (purple box), Tic20 domain (yellow box). The membrane-spanning models were predicted by the constrained consensus topology prediction (CCTOP) tools. Red wavy lines represent both the N- and C-termini facing the inner part of the membrane; blue wavy lines represent both the N- and C-termini facing the outer part of the membrane or the antipodal orientation of the N- and C-termini; and violet double horizontal lines represent the membrane lipid bilayer. Protein item accession numbers are shown in Table S1.

The structural features of Tic20 and Tic20-like proteins from different organisms were also annotated. The Tic20-like proteins can be roughly divided into three groups: the cyanobacteria group, the primary-plastid-possessing group (including some members of land plants, Rhodophyta and Chlorophyta), and the secondary-plastid-possessing group (including some members of Apicomplexa, Pyrrophyta, Chlorarachniophyta, Cryptophyta, and Stramenopiles) (Figure 2). The N-terminal BTS was found to exist exclusively in certain secondary-plastid-possessing organisms (Apicomplexa, Chlorarachniophyta, and Stramenopiles), and all the BTS-containing Tic20 proteins were predicted with the “W”-type membrane-spanning model. The N-terminal extra EF-hand domain also occurred in certain secondary-plastid-possessing organisms (Pyrrophyta and Stramenopiles) (Figure 2).

**Figure 3:**
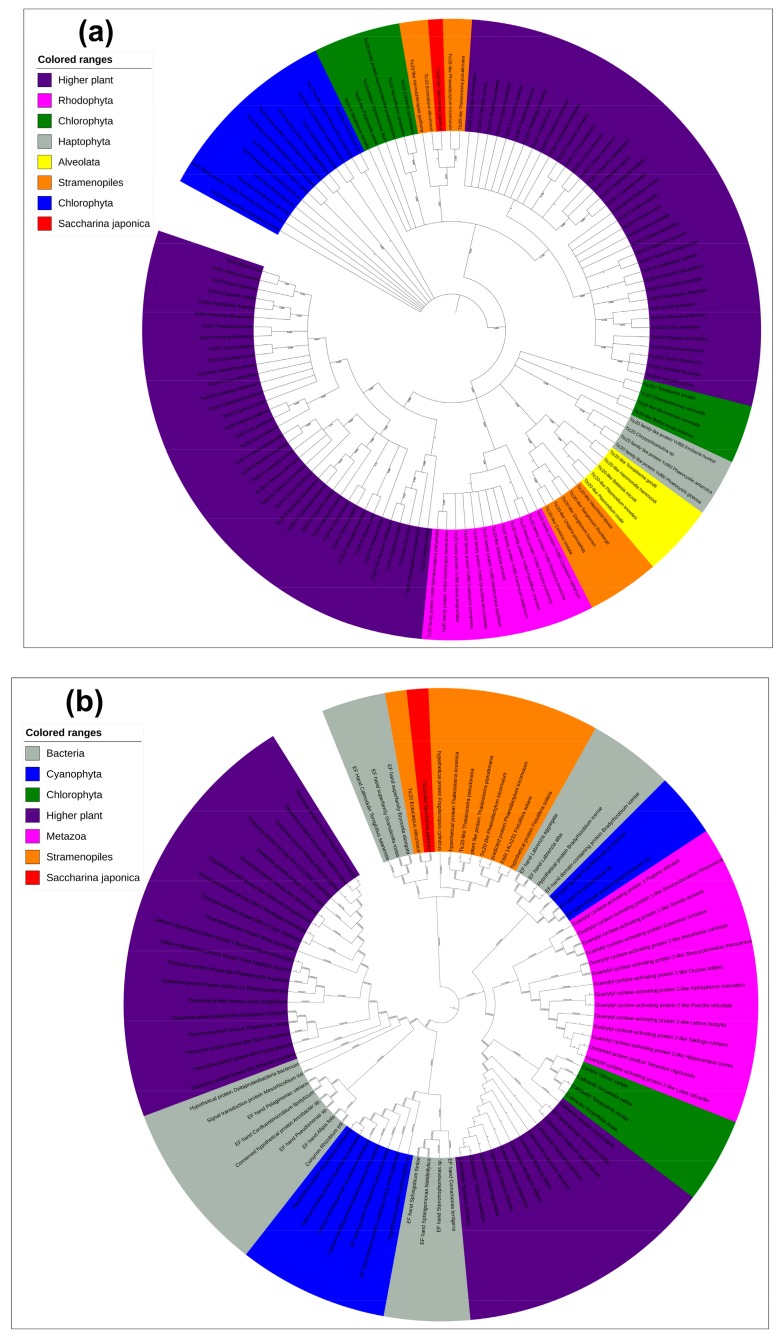
Phylogenetics of subsets of Tic20-like amino acids and EF-hand domains. (**a**) Phylogenetic analysis of Tic20-like proteins. Proteins from higher plants are shown in violet, Cyanophyta in blue, Chlorophyta in green, Rhodophyta in pink, Haptophyta in grey, Alveolata in yellow, Stramenopiles in orange, and *S. japonica* in red. (**b**) Phylogenetic analysis of EF-hand domain proteins. Proteins from bacteria are shown in grey, higher plants in violet, Metazoa in pink, Cyanophyta in blue, Chlorophyta in green, Stramenopiles in orange, and *S. japonica* in red. Protein item accession numbers are shown in Tables S2 and S3.

Phylogenetic analysis showed that the EF-hand domain (noncyanobacterium derived) and the Tic20 domain (cyanobacterium derived) had distinct evolutionary relationships (Figure 3) and that all the Tic20 domains were from cyanobacteria and could be divided into two distinct clades. The Tic20s from Rhodophyta, Haptophyta, Alveolata, some Chlorophyta and Stramenopiles, and higher plants formed one clade (Figure 3a). The other clade contained Tic20s from *S. japonica*, Chlorophyta, and other higher plants. All EF-hand domains exhibited signs of prokaryotic origin, and the EF-hand domains of higher plants could be divided into those that showed close relationships with metazoans, Chlorophyta and partial Cyanophyta and those that showed close relationships with other Cyanophyta. The EF-hand domains from Stamenopiles, including *S. japonica* are distant from those from higher plants, metazoan Chlorophyta, and Cyanophyta but exhibit close relationships with those from Acidobacteria (Figure 3b).

### 2.2. Localization of SjTic20-14 in P. Tricornutum

**Figure 4:**
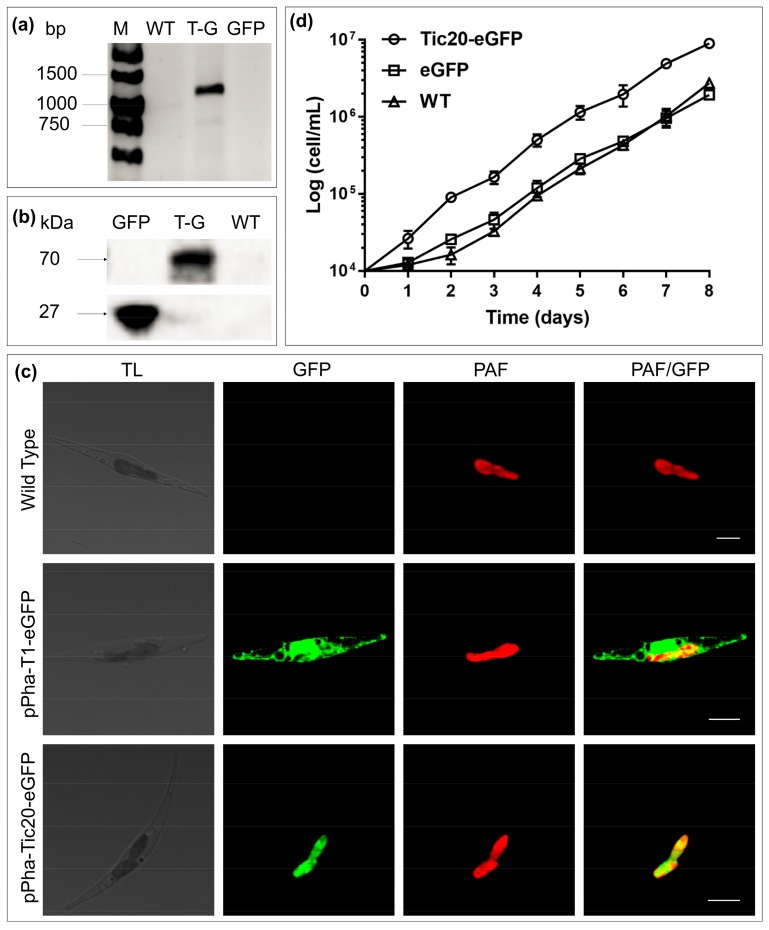
Transgenic diatom growth and observation of the fusion of SjTic20-14 and eGFP. (**a**) PCR detection of the SjTic20-eGFP sequence. WT: wild-type diatom, T-G: diatom expressing full-length SjTic20-eGFP; GFP: diatom expressing only eGFP (same below). (**b**) Total protein western blot for detection of the expression of eGFP: eGFP alone has a molecular weight of 26 kDa, and SjTic20-eGFP has a putative molecular weight of nearly 70 kDa. (**c**) Fluorescence laser scanning confocal microscopy observation. pPha-T1-eGFP: transformation of eGFP alone; pPha-Tic20-eGFP: transformation of SjTic20 fused to eGFP; TL: transit light; GFP: green fluorescence; PAF: plastid autofluorescence; PAF/GFP: overlay of plastid and green fluorescence; scale bar represents 5 μm. (**d**) Growth curve of diatom strains. Diatom strain expressing SjTic20-eGFP (curve with circles), wild-type strain (curve with triangles) and strain expressing eGFP alone (curve with squares).

When fused with enhanced green fluorescent protein (eGFP), SjTic20-14 exhibited green fluorescence signals in diatoms, which implied chloroplast localization (Figure 4; Figure S3a). The transgenic diatoms exhibited a significant increase in growth compared with the wild type (Figure 4d).

### 2.3. Transmembrane Topology of SjTic20-14 in P. Tricornutum

**Figure 5:**
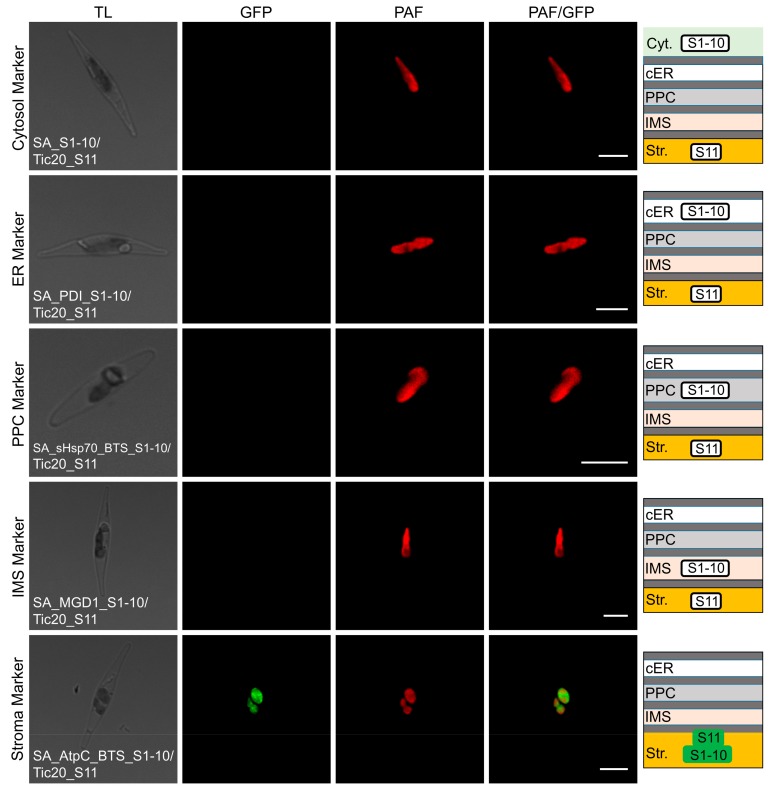
Self-assembling GFP assay analyzing the localization of full-length SjTic20-14 in *P. tricornutum*. SA_S1-10/Tic20_S11: co-expression of SjTic20-14 fused to GFP11 with the cytosol-localized GFP1-10; SA_PDI_S1-10/Tic20_S11: co-expression of SjTic20-14 fused to GFP11 with the ER-localized sequence fused to GFP1-10 (PDI_S1-10); SA_sHsp70_BTS_S1-10/Tic20_S11: co-expression of SjTic20-14 fused to GFP11 with the PPC-localized sequence fused to GFP1-10 (sHsp70_BTS_S1-10); SA_MGD1_S1-10/Tic20_S11: co-expression of SjTic20-14 fused to GFP11 with the IMS-localized sequence fused to GFP1-10 (MGD1_S1-10, same below); SA_AtpC_BTS_S1-10/Tic20_S11: co-expression of SjTic20-14 fused to GFP11 with the stroma-localized sequence fused to GFP1-10 (AtpC_BTS_S1-10, same below); Cyt.: cytosol; cER: chloroplast endoplasmic reticulum; PPC: periplastidial compartment; IMS: intermembrane space (same below); Str.: plastid stroma (same below); BTS: bipartite targeting sequence (same below); scale bar represents 5 μm.

To determine the subcellular location and topology of SjTic20-14 in the diatom, the self-assembling fragments of green fluorescent protein (GFP) assay was performed. For determination of the localization of the full-length protein, co-transformation of full-length SjTic20-14 with cytosol, endoplasmic reticulum (ER), periplastidial compartment (PPC), and intermembrane space (IMS) marker genes indicated no GFP signal emission, and the green fluorescent signal was only detected upon co-expression with the stroma marker gene, which confirmed the stromal location of the SjTic20-14 C-terminus (Table 1; Figure 5; Figure S3).

**Figure 6:**
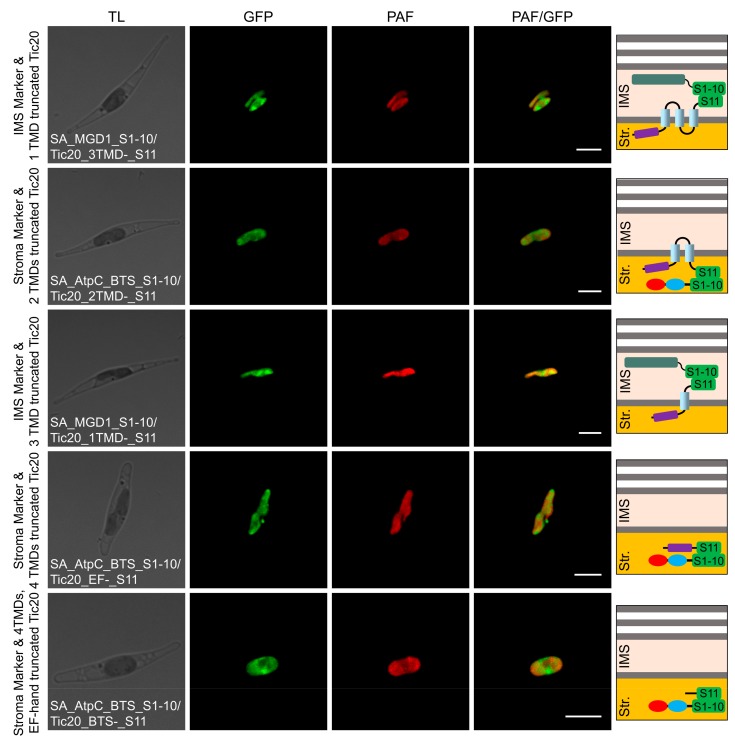
Self-assembling GFP assay analyzing the localization of domain-truncated versions of SjTic20-14 in *P. tricornutum*. SA_MGD1_S1-10/Tic20_3TMDs-_S11: co-expression of SjTic20-14 (with the last TMD truncated) fused to GFP11 with the IMS-localized sequence fused to GFP1-10; SA_AtpC_BTS_S1-10/Tic20_2TMDs-_S11: co-expression of SjTic20-14 (with the last 2 TMDs truncated) fused to GFP11 with the stroma-localized sequence fused to GFP1-10; SA_MGD1_S1-10/Tic20_1TMD-_S11: co-expression of SjTic20-14 (with the last 3 TMDs truncated) fused to GFP11 with the IMS-localized sequence fused to GFP1-10; SA_AtpC_BTS_S1-10/Tic20_EF-_S11: co-expression of SjTic20-14 (with all 4 TMDs truncated) fused to GFP11 with the stroma-localized sequence fused to GFP1-10; SA_AtpC_BTS_S1-10/Tic20_BTS-_S11: co-expression of SjTic20-14 (with all TMDs and the EF-hand domain truncated) fused to GFP11 with the stroma-localized sequence fused to GFP1-10; EF-: EF-hand domain; TMD: transmembrane domain; scale bar represents 5 μm.

In the localization analysis of the domain-truncated versions, green fluorescence signals revealed the IMS localization of the third TMD end, stromal localization of the second TMD end, IMS localization of the first TMD end, stromal localization of the EF-hand domain end, and stromal localization of the BTS end (Table 1; Figure 6; Figures S3 and S4). These results indicated that SjTic20-14 was localized in the innermost membrane of the *P. tricornutum* chloroplasts, with an N_in_-C_in_-terminal orientation and an “M”-like transmembrane model, and the EF-hand domain was entirely located in the stroma (Figure S5).

## 3. Discussion

As proteins need to cross the four membrane barriers of the secondary plastid, the corresponding preproteins generally carry an N-terminal BTS that constitutes an SP following a transit peptide-like (TPL) sequence [23]. Generally, the SP will be cut off by signal peptidase complex (SPC) after the preprotein crosses the outermost cER membrane, and the TPL sequence will be cleaved by the stromal processing peptidase (SPP) after the preprotein enters the stroma [2,12,23]. The SjTic20-14 protein also contains an N-terminal BTS, which may indicate the potential targeting of this protein to the chloroplast. Second, the cleavage sites between amino acids 21–22 and 58–59 may indicate that the first 58 amino acids undergo two cleavage processes to form the mature Tic20 protein. Further structural prediction revealed the existence of Tic20s with similar BTS structures in Apicomplexa, Stramenopiles, and some members of Chlorarachniophyta, showing that similar Tic20 transport machinery may be used in these organisms (Figure 2).

The EF-hand domain was proposed to be the most abundant domain superfamily, occurring extensively in proteins from distinct organisms [24]. Due to domain duplication, deletion, and shuffling, EF-hand domains have diverse structures and functions. A recent study divided the EF-hand superfamily into six groups composed of 156 subfamilies [24]. We used the EF-hand domain of SjTic20-14 as a query to run the blastp algorithm in NCBI, which showed that this domain shared the highest sequence similarity with the penta-EF-hand subfamily (PEF). As calcium sensors and calcium-dependent adaptors participate in diverse cellular activities, Ca^2+^ binding induces conformational changes in PEF domains, leading to the binding or dissociation of specific target proteins that play key roles in many processes, such as membrane trafficking, signal transduction, and endosomal biogenesis [25,26]. The existence of the N-terminal PEF-like EF-hand domain of SjTic20-14 implied the presence of a calcium-dependent channel in the chloroplast protein import pathway.

Interestingly, the noncanonical N-terminal EF-hand domain does not exist in higher plants. Further protein structural prediction of Tic20s from different taxa showed that EF-hand domains exist in red alga-derived plastids, including in Stramenopiles (Ochrophyta, Bacillariophyta) and dinoflagellates (Dinophyta) (Figure 2). Phylogenetic analysis showed that the Tic20 domain originated from cyanobacteria, and the EF-hand domain exhibited a noncyanobacterial origin. This phenomenon has been speculated to be caused by endosymbiotic gene transfer (EGT) [27], which could explain the origin of the noncanonical SjTic20-14 structure.

The subcellular localization of a protein is a key clue to its function. Reduced expression of chloroplast-localized AtTic20-I caused defects in preprotein import into chloroplasts and significant growth defects in *A. thaliana* [7,10,11]. Downregulated expression of the apicoplast-localized TgTic20 caused deficiency in growth and impairment of apicoplast preprotein import in *T. gondii* [15]. The chloroplast location of SjTic20-14 in diatoms and the obvious increase in the growth of SjTic20-14 transgenic diatoms compared with wild-type diatoms, implied that SjTic20-14 enhanced diatom growth. Combined with the observed association of 14 SjTic20 gene-containing regions with blade length and width in our early QTL mapping work [16], the expression of the SjTic20-14 gene alone gradually increased with *S. japonica* development (Figure S2). We infer that SjTic20-14 may also function in the chloroplast and influence the development of *S. japonica*. Therefore, the role of SjTic20-14 in the growth of *S. japonica*, especially in association with chloroplast protein import, should be explored in detail in future studies.

Transmembrane topology is crucial for the ability of proteins to perform diverse functions, such as cellular recognition, molecular receptor activity, passive and active transmembrane transport of substances, signal transduction, protein secretion, and enzymatic activity [14,18]. Although numerous computational programs for topology analysis are available, even the best algorithms to date can approach only 80% prediction accuracy [18]. We used diverse prediction tools to predict the topology of SjTic20-14 and obtained conflicting predictions of the “M” and “W” topologies, which will require further experimental verification (Figure S1d). Since the split nonfluorescent GFP fragments of β-strands 1-10 and β-strand 11 can spontaneously self-assemble to form fluorescent GFP within the same cellular compartment in vivo, a self-assembling GFP assay has been developed as a versatile tool to detect protein–protein interactions, folding, transport, and topology [28,29]. Our self-assembling assays co-expressed SjTic20-14 fragments with different domains truncated (0, 1, 2, 3, and 4 TMDs and all 4 TMDs plus the EF-hand domain) with fragments targeted to different subcompartments (cytosol, ER, PPC, IMS, and stroma) in the diatom. Green fluorescence showed the localization of SjTic20-14 to the innermost membrane of the chloroplast, with N_in_-C_in_-terminal stromal orientation and “M”-like transmembrane distribution. In addition, the entire EF-hand domain was present in the stroma. Despite the noncanonical EF-hand domain being fused with the Tic20 domain in SjTic20-14, the results of the subcellular localization and topology analyses were consistent with the findings for AtTic20-I in *A. thaliana* and TgTic20 in *T. gondii*. The conserved subcellular localization and topology of the Tic20s suggested that SjTic20-14 would exhibit similar subcellular localization and topology in *S. japonica*. Furthermore, the fusion of the N-terminal calcium-sensing EF-hand domain and the C-terminal Tic20 translocon domain indicated that translocon activity may be modulated by the binding of Ca^2+^ to the EF-hand domain, which requires further experimental verification.

## 4. Materials and Methods

### 4.1. 5’- and 3’-RACE of the Candidate Tic20 Sequence

The CDSs of the 14 *SjTic20* genes (GenBank ID: KY411547-KY411560) were predicted with GeneScan (http://www.techdragon.com.hk/documents/genescan.html). The CDSs were subjected to a BLAST search against previous transcriptomic data (BioProject ID: PRJNA512328), and the best-fit reads were identified as *Tic20* candidate sequences for further 5’- and 3’-RACE.

*S. japonica* (strain 860) juvenile sporophytes were collected and prepared according to previously described methods [16]. Total RNA was extracted with the Plant RNA Kit (Omega Bio-Tek, Norcross, GA, USA), and the extracted RNA was used to generate a 5’/3’-RACE read with the SMARTer RACE 5’/3’ Kit (Clontech, Mountain View, CA, USA). The first-strand cDNA was synthesized with the PrimeScript RT Reagent Kit (Takara, Tokyo, Japan), and the primers were designed based on *SjTic20* candidate sequences (Table S4).

To quantitatively confirm the presence of *SjTic20* in *S. japonica* at different developmental stages, we used the SYBR Premix Ex Taq (TaKaRa, Tokyo, Japan) system for amplification verification on a TaKaRa TP800 thermal system (TaKaRa, Tokyo, Japan).

### 4.2. Prediction of Domain Architecture and Sequence Features

The full-length CDSs were predicted by ORF Finder (https://www.ncbi.nlm.nih.gov/orffinder/), and the basic physical and chemical parameters of the putative protein were calculated by ProtParam (http://web.expasy.org/protparam/). PredictProtein (https://www.predictprotein.org/) was combined with PFAM (http://pfam.sanger.ac.uk) and InterProScan (http://www.ebi.ac.uk/tools/interproscan) for motif and domain analyses. SP and TP sequences were analyzed by SignalP 4.1 (http://www.cbs.dtu.dk/services/SignalP/) and ChloroP1.1 (http://www.cbs.dtu.dk/services/ChloroP/), respectively. Protein subcellular localization was predicted by TargetP (http://www.cbs.dtu.dk/services/TargetP). The online CCTOP (http://cctop.enzim.ttk.mta.hu/?_=/jobs/submit) tool was used to identify the SjTic20 transmembrane topology [30]. Phylogenetic analysis by the neighbor-joining method was performed using MEGA 7.0 [31], and the annotation was conducted with iTOL software (http://itol.embl.de/itol.cgi).

### 4.3. Culture of P. Tricornutum

*P. tricornutum* (strain 18.6) was cultured at 19 °C in f/2 medium under continuous illumination (150 μmol photons m^−2^·s^−1^) [32].

### 4.4. eGFP Localization and Self-Assembling GFP Assay

All vectors were constructed with the Gibson Assembly Master Mix Kit (New England Biolabs, Beverly, MA, USA) according to the same overlapping sequences between the ends of adjacent fragments; the primers used are listed in Table S4. The *egfp* region was inserted into the 3’-end of the complete *SjTic20* CDS (without the terminal codon) and was integrated into the pPha-T1 vector (NCBI accession no. AF219942). The pPha-T1 vector with only *egfp* inserted was regarded as the positive control vector. All the vectors were transformed into *P. tricornutum* with the Biolistic PDS-1000/He particle delivery system (Bio-Rad, Hercules, CA, USA), according to previously reported methods [32,33].

The self-assembling GFP assay method was used for subcellular localization and topological analyses [28]. The *P. tricornutum* transformation vector pPha_DUAL_2xNR (NCBI accession no. JN180664) was used to construct the expression plasmids [34]. The linearized vector fragment from the first multiple cloning site (MCSI) was generated by cleavage at the *Spe*I and *Sac*II restriction sites; a linearized vector fragment from the second multiple cloning site (MCSII) was generated by cleavage at the *Eco*RI and *Hind*III restriction sites. All *SjTic20*, *gfp1-10*, and *gfp11* fragments were amplified using primers equipped with terminal overlapping sequences (Table S4). The SjTic20 regions encoding the first 412, 358, 322, 282, 250, and 100 aa (corresponding to deletion of 0, 1, 2, 3, and 4 of the protein TMDs and of all 4 TMDs plus the EF-hand domain, respectively), with the 3’-end fused to the small *gfp* sequence (GFP11), were cloned into MCSII. For the MCSI insertion, 4 marker genes with 3’-ends were combined with the *gfp* sequence (GFP1-10), and one additional GFP1-10 was inserted solely as a cytosol localization marker. The 4 marker genes used were the ER-localized protein of disulphide isomerase (PDI) [35], the PPC-localized BTS of heat shock protein 70 (Hsp70) [35], the IMS-localized protein of mono-galactosyldiacylglycerol synthase 1 (MGD1) [36] and the stroma-localized BTS of ATPase gamma subunit (AtpC) [35]. All vector transformations were conducted using the protocols described above.

### 4.5. PCR Analysis, Western Blotting, and Flow Cytometry Detection

After 3–4 weeks of resistance screening on solid f/2 plates containing 100 μg/mL Zeocin, the colonies were selected and transferred into 10 μL Nonidet P 40 (NP-40), incubated at 95 °C for 10 min. The lysate samples were placed on ice for 10 min and diluted to 100 μL with ddH_2_O. Then, 5 μL aliquots of the diluted samples were used as templates for PCR detection with gene-specific primers (Table S4).

The positive Tic20-eGFP transgenic colonies were inoculated into 30 mL of f/2 liquid medium containing 100 μg/mL phleomycin. After one week of growth, the diatoms were harvested by centrifugation (3,000 rpm, 5 min) and resuspended in 300 μL of lysis buffer (50 mM Tris-HCl (pH 6.8), 2% SDS) with a protease inhibitor cocktail (1:25). The supernatant was extracted after incubation at room temperature (RT) for 30 min, followed by centrifugation at 4 °C for 30 min at 13,000 rpm. The proteins were separated on an 8% polyacrylamide gel (SDS-PAGE). Next, western blot analysis was conducted, and immunodetection was performed using monoclonal antibodies at a dilution of 1:2500 against GFP (TRAN, Beijing, China). The fluorescence signals of all the transgenic diatom strains were quantified in a BD FACSII flow cytometry system (BD, Franklin Lake, NJ, USA).

### 4.6. Growth Rate Measurements

*P. tricornutum* wild-type, eGFP, and Tic20-eGFP transgenic cell lines (three biological duplication samples per cell line) were cultivated under normal conditions (the culture media of the transgenic cell lines were supplemented with 100 μg/mL Zeocin) to the exponential phase. After 12 h of darkness to arrest the cells in G1 for synchronization [37], each cell line was diluted to the same concentration (5 × 10^4^ cells/mL) and grown under normal conditions with light. Cell densities were measured by flow cytometry every day for 8 consecutive days.

### 4.7. Laser Scanning Confocal Microscopic Observation

Positive transgenic *P. tricornutum* colonies were inoculated into f/2 liquid medium containing 100 μg/mL Zeocin for one week of growth. The green fluorescence signals were observed and recorded under a ZEISS LSM710 laser scanning confocal microscope (Carl Zeiss, Jena, Germany) with an excitation wavelength of 488 nm and emission wavelength of 510–540 nm for eGFP and 625–720 nm for chloroplast autofluorescence.

## 5. Conclusions

In summary, we characterized SjTic20-14 and found a noncanonical N-terminal EF-hand domain of noncyanobacterial origin fused to the C-terminal domain of cyanobacterial origin within the Tic20 domain structure. The protein localized to the innermost membrane of the chloroplast, exhibited an N_in_-C_in_-terminal stromal orientation and contained four TMDs in an “M”-like configuration. Transgenic SjTic20-14 enhanced diatom growth, and the association of this complex with the growth of *S. japonica* needs to be explored in detail.

## Figures and Tables

**Table 1:** Statistical results of the self-assembling GFP assay.

SjTic20-14 Fragments	GFP Signal Detection					Localization
	Cytosol	cER	PPC	IMS	Stroma	
Full-length (0 TMDs deleted)	− ^1^	−	−	−	+ ^2^	Stroma
Last 1 TMD deleted	−	−	−	+	−	IMS
Last 2 TMDs deleted	−	−	−	−	+	Stroma
Last 3 TMDs deleted	−	−	−	+	−	IMS
All 4 TMDs deleted	−	−	−	−	+	Stroma
All TMDs and EF−hand domain deleted	−	−	−	−	+	Stroma

^1^ “−” indicates that no green fluorescence signal was detected for the SjTic20-14 fragment co-expressed with the corresponding cytosol, cER, PPC, IMS, and stroma marker genes; ^2^ “+” indicates that a green fluorescence signal was detected for the SjTic20-14 fragment co-expressed with the corresponding marker genes.

## Supplementary Materials

Supplementary materials can be found at https://www.mdpi.com/1422-0067/20/16/4000/s1.

## Abbreviations

AtpC	ATPase gamma subunit
BTS	Bipartite targeting sequence
ER	Endoplasmic reticulum
Hsp70	Heat shock protein 70
IMS	Intermembrane space
MGD1	Mono-galactosyldiacylglycerol synthase 1
PDI	Protein disulphide isomerase
PPC	Periplastidial compartment
SP	Signal peptide
TIC	Translocon at the inner envelope membrane of the chloroplast
TMD	Transmembrane domain
TOC	Translocon at the outer envelope membrane of the chloroplast
TP	Transit peptide
