# Supplementary material for: Verification of the Saccharina japonica Translocon Tic20 and its Localization in the Chloroplast Membrane in Diatoms
Source: Int J Mol Sci. 2019 Aug 16;20(16):4000. doi: 10.3390/ijms20164000 (PMC6720183; doi:10.3390/ijms20164000)
Supplement: Supplementary file 1 [file ijms-20-04000-s001.zip › Supplementary matierals/Supplementary Figures S1-S5.docx]

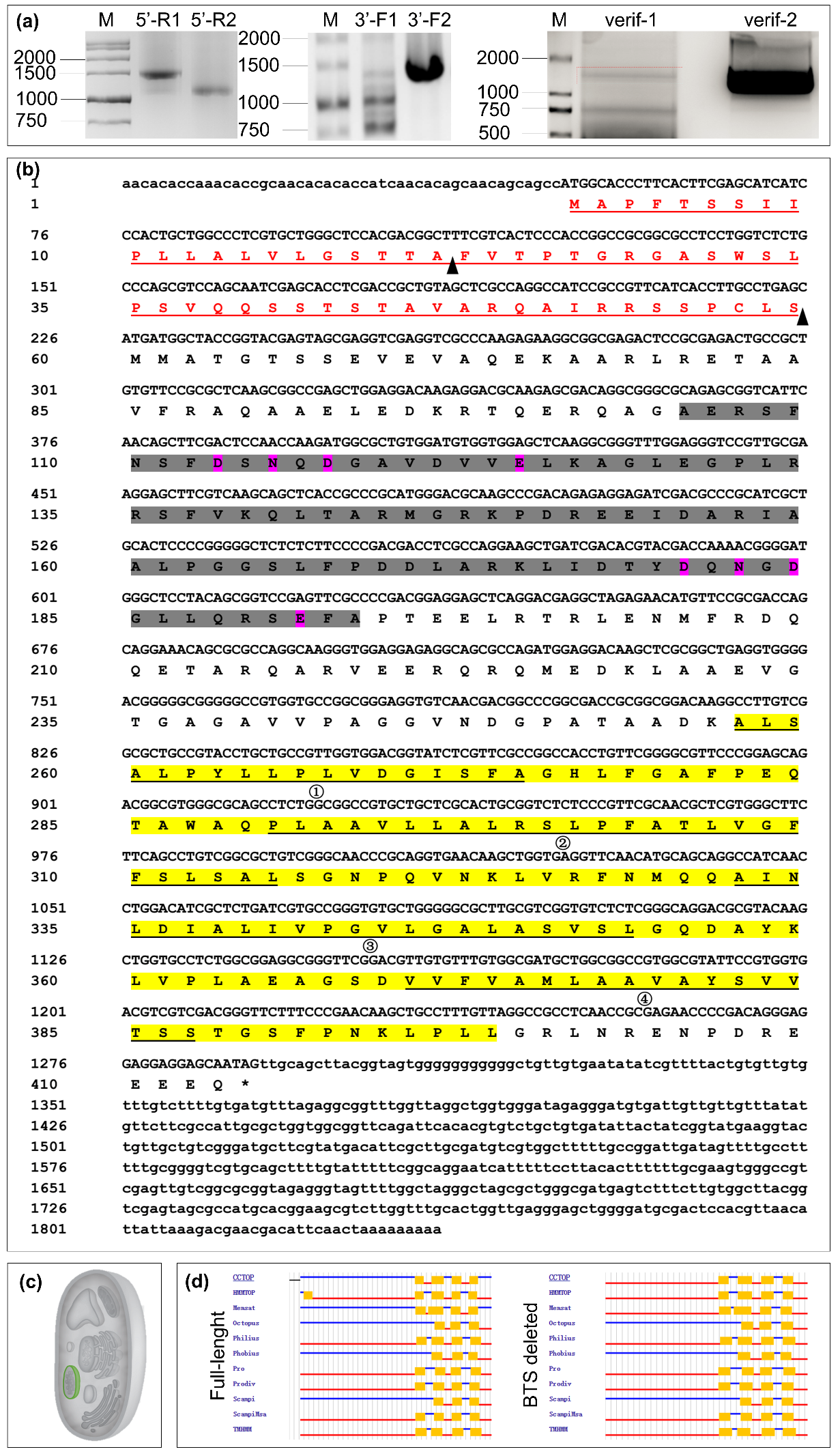


**Figure S1.** *SjTic20-14* gene clone and the obtained nucleotide sequence with the feature of deduced amino acids. (**a**) Gene clone and verification electrophoretograms. The gained 5’ unknown fragments with Tic-5R1/UPM primer pairs (5’-R1) and Tic-5R2/UPS primer pairs (5’-R2) respectively in the left picture; the gained 3’ unknown fragments with Tic-3F1/UPM primer pairs (3’-R1) and Tic-3F2/UPS primer pairs (3’-F2) respectively in the middle picture; fragment verification of the 5’/3’-unknown sequence assembling was checked by RT-PCR using Tic-F1/Tic-R1 primer pair (verif-1) and nest PCR using Tic-F2/Tic-R2 primer pair (verif-2) in the right picture. (**b**) *SjTic20-14* nucleotide sequence and the open reading frame deduced amino acids. Bipartite sequences (signal peptide and transit peptide in red, underline), cleavage sites (black triangle), EF-hand superfamily domain (grey shadow), Ca^2+^ binding site (pink shadow), Tic20 superfamily (yellow shadow), transmembrane regions (underline with circler number below to represent the sorts of TMDs). (**c**) Subcellular localizational prediction of SjTic20-14 (the predicted subcellular localization area colored in green). (**d**) Ambiguous transmembrane topology prediction results of SjTic20-14 (yellow boxes indicated transmembrane domains, membrane inner part colored in red line and outer part colored in blue line; left: topology prediction of SjTic20-14 full-length; right: topology prediction of SjTic20-14 with BTS deleted).


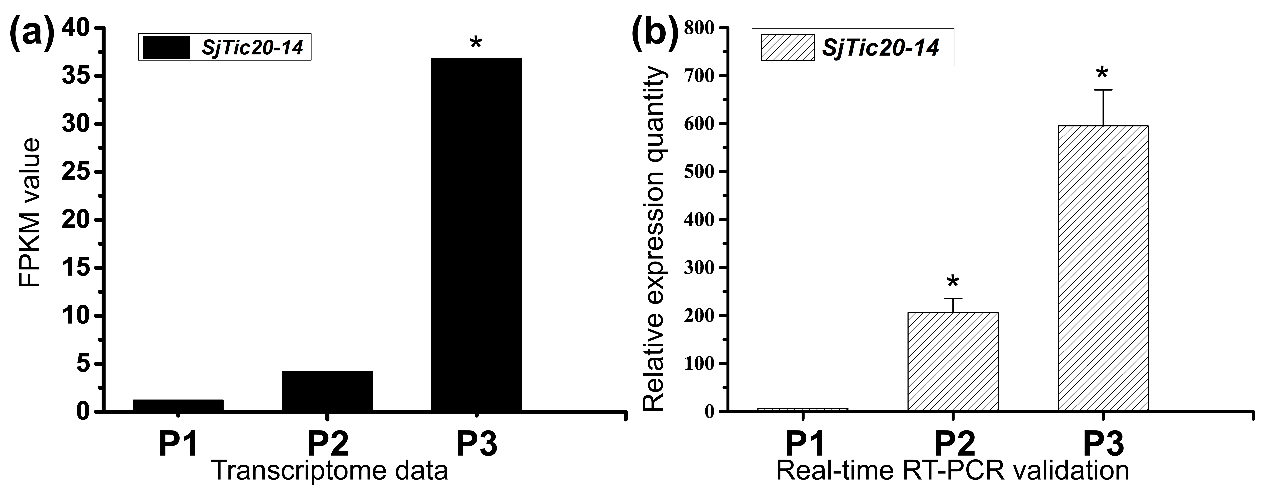


**Figure S2.** Expression level of *SjTic20-14* in 3 developmental periods. (**a**) Transcriptome FPKM value of the matched read represent the expression level of *SjTic20-14*. (**b**) *SjTic20-14* qPCR analysis validation. P1, P2, P3: developmental period (sampled in April 16th, May 6th, June 2th 2015 respectively).


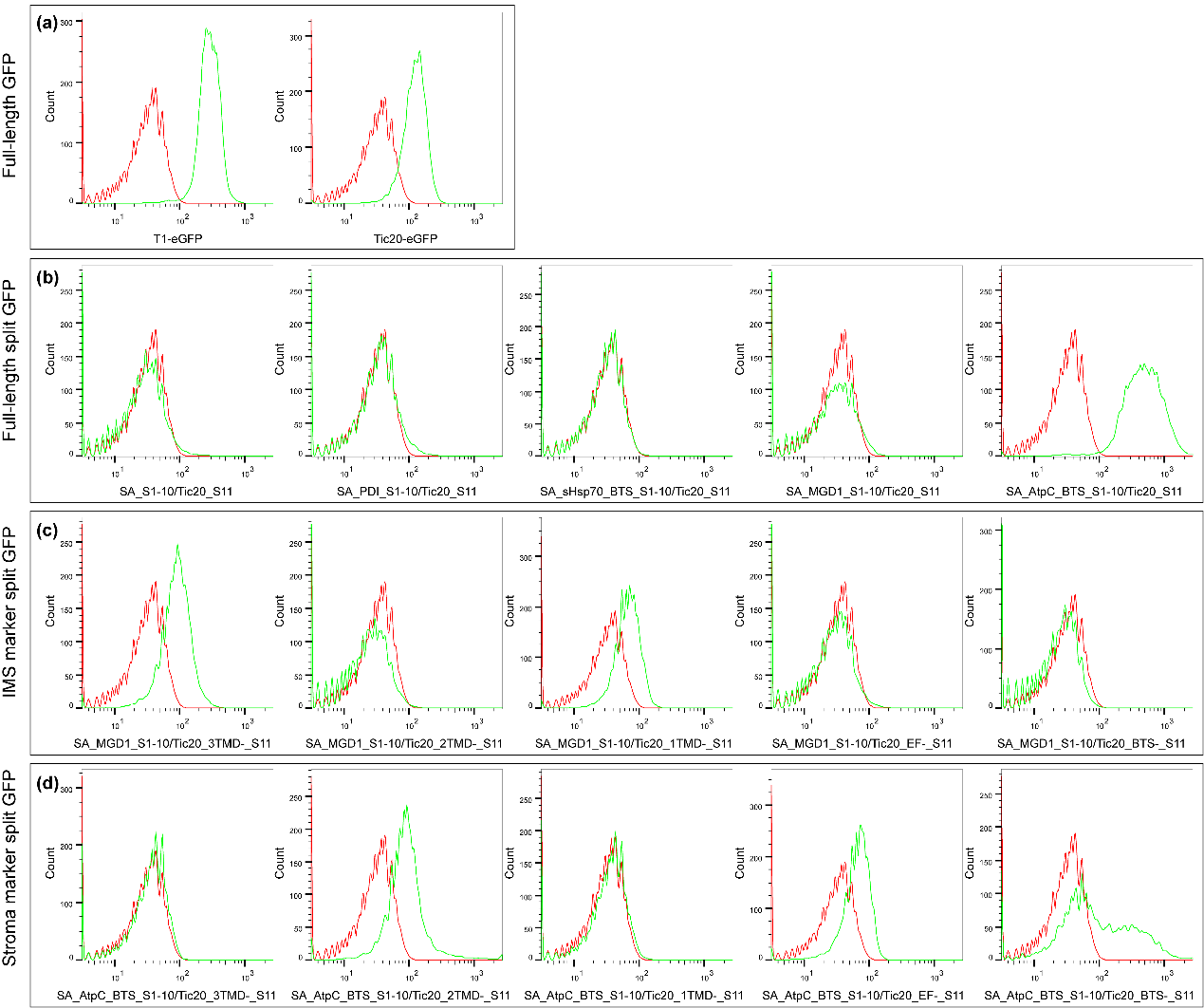


**Figure S3.** Green fluorescent quantification of transformed diatoms. To qualify fluorescence levels, all transfected diatom cell strains were analyzed by flow cytometry. Fluorescence intensity of every transforming diatom cell strain (presenting by green line in every diagrams) were merged with the fluorescence intensity of wild sample (red line). (**a**) The full-length eGFP assays. T1-eGFP: fluorescence intensity in full-length eGFP; Tic20-eGFP: full-length Tic20 fused eGFP; green fluorescence are considerably greater than the intensity in wild type, exhibiting positive signals. (**b**) The full-length split GFP assays. SA_S1-10/Tic20_S11: co-expression of Tic20-14 with cytosol marker gene; SA_PDI_S1-10/Tic20_S11: co-expression of Tic20-14 with cER marker gene; SA_sHsp70_BTS_S1-10/Tic20_S11: co-expression of Tic20-14 with PPC marker gene; SA_MGD1_S1-10/Tic20_S11: co-expression of Tic20-14 with IMS marker gene; SA_AtpC_BTS_S1-10/Tic20_S11: co-expression of Tic20-14 with stroma marker gene. Positive signal can be detected in co-expression of Tic20_S11 with AtpC_BTS_S1-10 means the stroma localization of the Tic20 C-terminus. (**c**) The Tic20 domains truncated sequences co-expression with IMS marker gene. SA_MGD1_S1-10/Tic20_3TMD-_S11: co-expression of IMS marker gene with the last TMD truncated Tic20-14; SA_MGD1_S1-10/Tic20_2TMD-_S11: co-expression with the last 2 TMDs truncated Tic20-14; SA_MGD1_S1-10/Tic20_1TMD-_S11: co-expression with the last 3 TMDs truncated Tic20-14; SA_MGD1_S1-10/Tic20_EF-_S11: co-expression with the all 4 TMDs truncated Tic20-14; SA_MGD1_S1-10/Tic20_BTS-_S11: co-expression with the BTS sequence of Tic20-14. Positive signals existing in con-transforming of IMS marker gene with Tic20_3TMD-_S11 and Tic20_1TMD-_S11 means the IMS localization of the 1^st^ and 3^rd^ TMD-terminus. (**d**) The Tic20 domains truncated sequences co-expression with stroma marker gene. SA_AtpC_BTS_S1-10/Tic20_3TMD-_S11: co-expression of stroma marker gene with the last TMD truncated Tic20-14; SA_AtpC_BTS_S1-10/Tic20_2TMD-_S11: co-expression with the last 2 TMDs truncated Tic20-14; SA_AtpC_BTS_S1-10/Tic20_1TMD-_S11: co-expression with the last 3 TMDs truncated Tic20-14; SA_AtpC_BTS_S1-10/Tic20_EF-_S11: co-expression with the all 4 TMDs truncated Tic20-14; SA_AtpC_BTS_S1-10/Tic20_BTS-_S11: co-expression with the BTS sequence of Tic20-14. Positive signals existing in con-transforming of IMS marker gene with Tic20_2TMD-_S11, Tic20_EF-_S11 and Tic20_BTS-_S11 means the stroma localization of the 2^nd^ TMD, EF-hand domain and the BTS C-terminus. Together, the split GFP assays (**b**, **c**, and **d**) exhibiting the SjTic20-14 chloroplast innermost membrane localization, N_in_-C_in_ terminus stromal orientation, “M” like membrane spanning model and the EF-hand domain entirely in the stroma compartment.


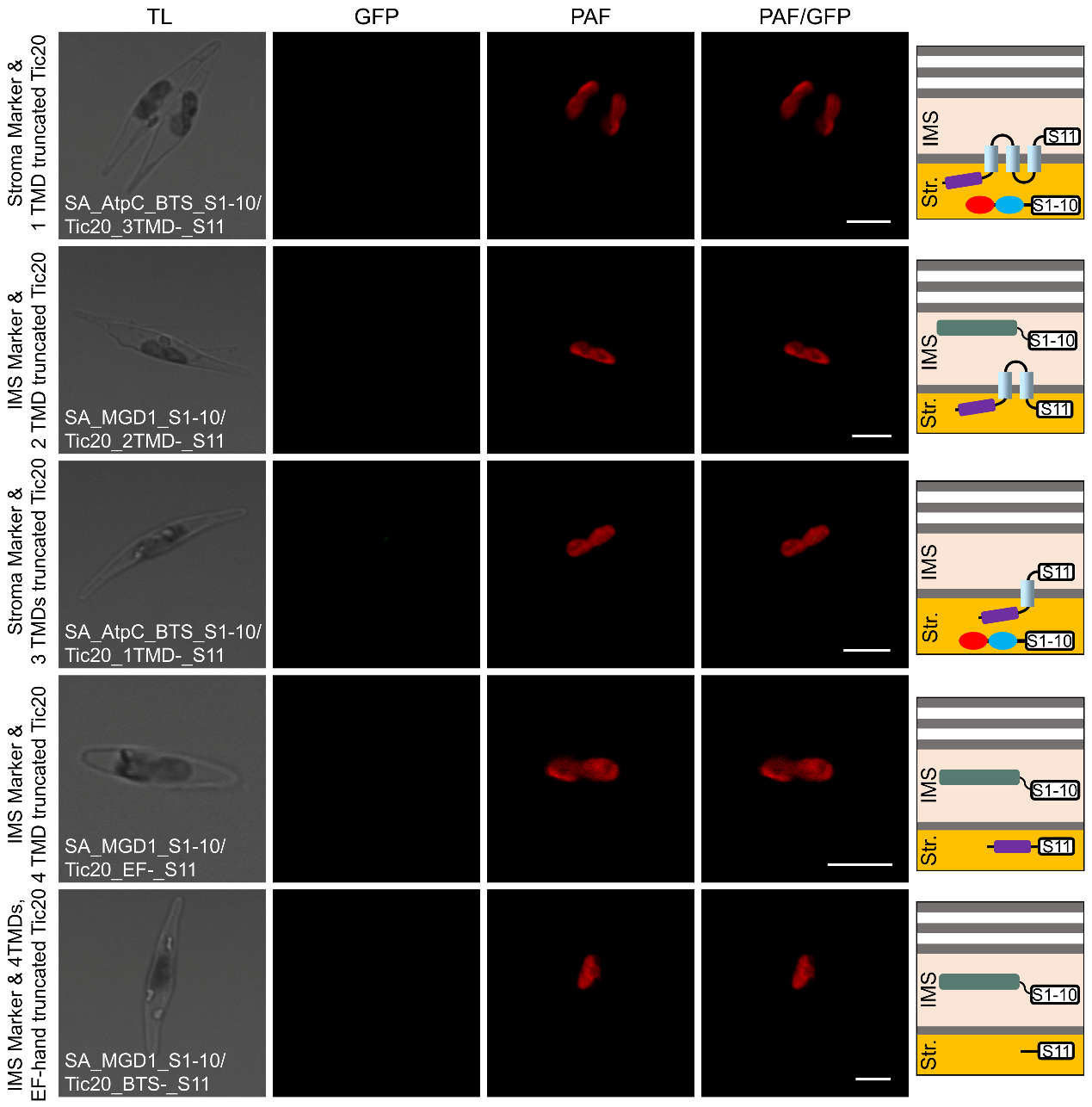


**Figure S4.** Self-assembling GFP assay analyzing the localization of different domains truncated SjTic20-14 in *P. tricornutum*. SA_AtpC_BTS_S1-10/Tic20_3TMDs-_S11: co-expression of SjTic20-14 (with the last 1 TMD truncated) fused to GFP11 with the stromal localized sequence fused to GFP1-10; SA_MGD1_S1-10/Tic20_2TMDs-_S11: co-expression of SjTic20-14 (with the last 2 TMDs truncated) fused to GFP11 with the IMS localized sequence fused to GFP1-10; SA_AtpC_BTS_S1-10/Tic20_1TMDs-_S11: co-expression of SjTic20-14 (with the last 3 TMDs truncated) fused to GFP11 with the stromal localized sequence fused to GFP1-10; SA_MGD1_S1-10/Tic20_EF-_S11: co-expression of SjTic20-14 (with the all 4 TMDs truncated) fused to GFP11 with the IMS localized sequence fused to GFP1-10; SA_MGD1_S1-10/Tic20_EF-_S11: co-expression of SjTic20-14 (with all TMDs and EF-hand domain truncated) fused to GFP11 with the IMS localized sequence fused to GFP1-10; TMD: transmembrane domain; EF-: EF-hand domain; scale bar represents 5 μm.


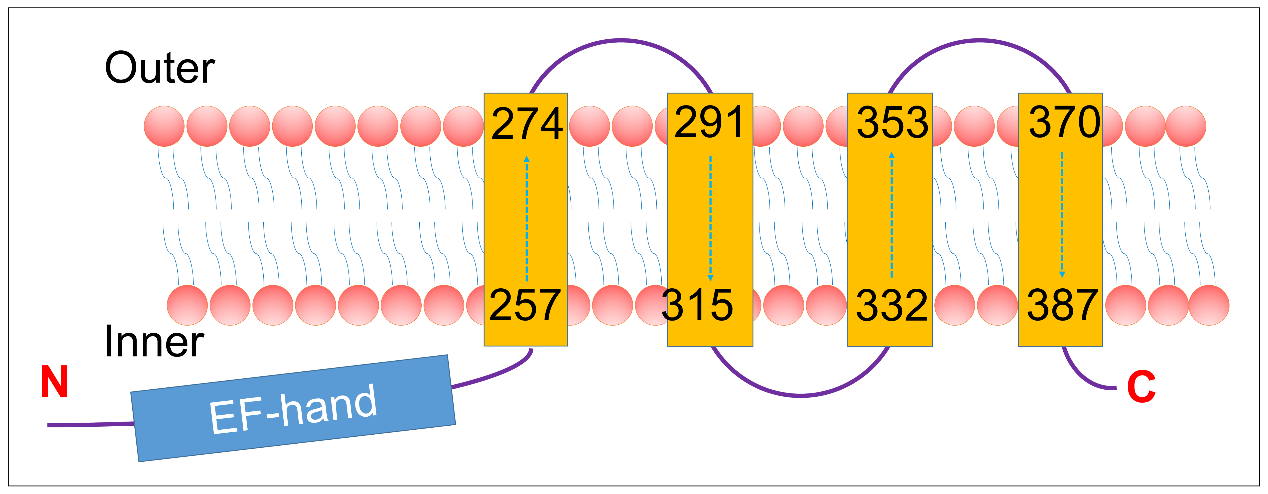


**Figure S5.** Schematic illustration of structure and membrane spanning model of predicted mature *Sj*Tic20-14 protein. The pink circles constructed 2 lines represent chloroplast innermost membrane bilayers; “Outer” represent the IMS compartment; “Inner” represent the chloroplast stroma; EF-hand domain represented by blue rectangle; TMDs in the Tic20 domain represented by yellow rectangle, numbers at the 2 terminal borders represent the predicted TMD’s start and stop sites, and blue dots arrow represent the TMD’s transmembrane orientation.
